# Global burden of colorectal cancer and associated risk factors in young-older adults: Trends from 1990 to 2021 with projections to 2050

**DOI:** 10.1097/MD.0000000000047448

**Published:** 2026-02-06

**Authors:** Cailu Shen, Caier Cai, Ying Zhang, Hejia Xu, You Wu, Tong Xu, Yong Mao

**Affiliations:** a Department of Cancer Diagnosis and Treatment Center, Affiliated Hospital of Jiangnan University, Wuxi, China; b Wuxi Medical College of Jiangnan University, Wuxi, China.

**Keywords:** colorectal cancer, global burden of disease, projections, risk factors, young-old adults

## Abstract

Colorectal cancer (CRC) poses a significant global health challenge, yet the disease burden and epidemiological trends in young-older adults (65–74 years) remain poorly understood. This age group warrants special attention because it experiences peak disease incidence while maintaining relatively preserved functional capacity and better tolerance to therapy compared with individuals aged ≥75 years. Data from the Global Burden of Disease 2021 were used to assess the CRC burden in individuals aged 65 to 74 years from 1990 to 2021. Age-standardized rates for incidence, prevalence, mortality, and disability-adjusted life years were computed, alongside temporal trend analysis via estimated annual percentage change (EAPC). We also evaluated disease burden associations with socio-demographic index, burden trends from major risk factors, and disease projections to 2050 using autoregressive integrated moving average modeling. Globally, age-standardized rates for incidence increased modestly from 125.03 per 100,000 person-years in 1990 to 136.19 per 100,000 person-years in 2021 (EAPC 0.10%, 95% confidence interval: 0.03–0.16%). Males demonstrated increasing trends (EAPC 0.39%) while females showed declining patterns (EAPC −0.36%). Middle-income countries and East Asia exhibited steepest increases (EAPC 1.68% and 1.99%), contrasting with reductions in high-income regions. Age-standardized mortality rate declined significantly from 73.41 per 100,000 person-years in 1990 to 58.48 per 100,000 person-years in 2021 (EAPC −0.96%). High-income regions achieved superior mortality improvements, particularly Australasia (EAPC −2.72%) and North America (EAPC −2.32%). Diet low in milk constituted the leading attributable risk factor in 2021. Projections indicate continued mortality decline (EAPC −0.80%) alongside persistent prevalence growth (EAPC 0.57%) through 2050. Among young-older adults, CRC burden demonstrates declining mortality alongside rising incidence and prevalence, with substantial regional inequalities. Targeted interventions addressing modifiable risk factors are essential, particularly in middle-income regions undergoing epidemiological transition.

## 1. Introduction

Colorectal cancer (CRC) ranks 3rd among global cancers and 2nd in cancer-related mortality worldwide, with 1.9 million new cases and over 0.9 million deaths recorded in 2020, projected to reach 3.2 million cases by 2040.^[1–3]^ While developed nations maintain higher incidence rates, emerging economies show accelerating trends reflecting lifestyle transitions and demographic changes.^[4]^ Current epidemiological patterns present complexity: new diagnoses have stabilized among adults ≥50 years in high-income countries, yet older populations face the greatest disease impact, with individuals ≥65 years representing approximately 60% of all cases.^[5–7]^

Adults aged 65 to 74 years (termed “young-older adults”) present compelling reasons for focused investigation.^[8]^ This population experiences peak disease occurrence while maintaining relatively preserved functional capacity and better therapeutic tolerance compared to individuals ≥75 years, presenting important intervention opportunities.^[9]^ Recent clinical trials have validated colonoscopy effectiveness for mortality reduction in this age stratum, while lower competing health risks enhance the cost-effectiveness of preventive measures.^[10,11]^ Moreover, socioeconomic development patterns significantly influence disease distribution through healthcare accessibility, nutritional transitions, and behavioral risk factor prevalence, creating substantial variation across regions with different development trajectories.

Despite this population’s clinical importance, significant research gaps persist. Recent Global Burden of Disease (GBD) analyses have documented CRC trends globally and regionally.^[12–16]^ However, these investigations exhibit notable limitations: some analyze broader age categories without specifically examining the 65 to 74 age bracket,^[12,14,15]^ others lack systematic risk factor quantification,^[13,16]^ and several provide projections extending only to 2040 or earlier.^[13,15]^ Critically, age-specific investigation of the 65 to 74 demographic is essential because this group represents a unique convergence of peak disease incidence, preserved intervention capacity, and distinct risk factor profiles shaped by historical cohort exposures (characteristics that broader age categorizations obscure).

To address these gaps, we conducted a comprehensive global assessment of CRC burden among individuals aged 65 to 74 years using GBD 2021 data. Our study integrated 4 analytical dimensions: age-specific burden quantification across incidence, prevalence, mortality, and disability-adjusted life years (DALYs); systematic attribution of burden to 10 modifiable risk factors spanning metabolic, dietary, and lifestyle factors; frontier analysis benchmarking national performance against optimal achievable outcomes; and validated projections to 2050 using autoregressive integrated moving average (ARIMA) modeling. We examined temporal trends from 1990 to 2021 across multiple analytical scales (global, regional (21 GBD regions and 5 socio-demographic index [SDI] quintiles), and national (204 countries and territories)) to inform evidence-based prevention strategies and optimize resource allocation for this high-burden yet intervention-responsive population.

## 2. Materials and methods

### 2.1. Data source and collection

Data were extracted from the GBD 2021 study, maintained by the Institute for Health Metrics and Evaluation, accessed via the Global Health Data Exchange (https://ghdx.healthdata.org/gbd-results-tool). The GBD 2021 provides comprehensive assessment of health risks related to 371 diseases and injuries and 88 risk factors, employing unified methodology for multi-indicator estimation across 204 countries and territories.^[17]^ Countries were stratified into 21 GBD regions and 5 SDI quintiles (low, low-middle, middle, high-middle, and high).^[18]^ The SDI represents a composite metric calculated as the geometric mean of lag-distributed income per capita, average educational attainment among individuals aged ≥15 years, and total fertility rate among women under 25 years.^[19]^ We extracted CRC burden metrics (including incidence, prevalence, mortality, and DALYs) for individuals aged 65 to 74 years, with corresponding 95% uncertainty intervals (UIs) derived from the 2.5th and 97.5th percentiles of the posterior distribution, covering the period 1990 to 2021. Analyses were stratified by sex and geographic levels. We quantified CRC mortality and DALYs attributable to major modifiable risk factors: metabolic (high body mass index [BMI], high fasting plasma glucose), dietary (diet low in milk, high in red meat, high in processed meat, low in fiber, low in calcium), and lifestyle factors (smoking, low physical activity, high alcohol use).^[20]^ Population data through 2050 were obtained from UN World Population Prospects.^[21]^

### 2.2. Ethics statement

This study was exempted from ethical review by the Ethics Committee of Affiliated Hospital of Jiangnan University, as it involved secondary analysis of publicly available aggregated data. The GBD 2021 study was ethically approved by the Institutional Review Board at the University of Washington. No individual-level or personally identifiable information was accessed or analyzed.

### 2.3. Definition

CRC is diagnosed through endoscopy, histopathological biopsy, and cytological evaluation based on relevant clinical signs and symptoms. According to the International Classification of Diseases (ICD), CRC is defined by ICD-9 codes 153.0 to 154.9, 209.1 to 209.17, V10.05 to V10.06, and V76.41 to V76.52, or ICD-10 codes C18 to C19.0, C20, and C21 to C21.8.^[22]^ Four standardized epidemiological metrics were employed to characterize temporal patterns in CRC disease burden: the age-standardized incidence rate (ASIR), quantifying newly diagnosed cases per 100,000 person-years; the age-standardized prevalence rate (ASPR), denoting the proportion of individuals living with CRC per 100,000 population at a specified time point; the age-standardized mortality rate (ASMR), representing CRC-attributable deaths per 100,000 person-years; and the age-standardized DALYs rate (ASDR), measuring disease burden as years of healthy life lost per 100,000 population. To ensure cross-regional comparability and account for demographic heterogeneity, all rates underwent age standardization using the GBD 2019 reference population through direct standardization methodology, which adjusts observed data to a standardized population structure.^[23]^

### 2.4. Data analysis

The age-standardized rates were calculated as:


$$\begin{array}{l} \begin{array}{l} \;ASR=\left(\frac{\overset{A}{\underset{i=1}{\sum }}\left(\alpha_{i\;}\times w_{i}\right)}{\overset{A}{\underset{i=1}{\sum }}w_{i}}\right)\times 100,000\#\; \end{array} \end{array}$$

where $\alpha_{i}$ is the rate in the $i_{th}$ age group and $w_{i}$ is the GBD standard population number in the $i_{th}$ age group. Longitudinal trends in ASIR, ASPR, ASMR, and ASDR spanning the period 1990 to 2021 were quantified using estimated annual percentage changes (EAPCs) with their corresponding 95% confidence intervals (CIs). The analytical approach employed linear regression modeling:


$$\begin{array}{l} \begin{array}{l} y=\alpha +\beta T+\varepsilon \#\; \end{array} \end{array}$$

$$\begin{array}{l} \begin{array}{l} EAPC=100\times \left(exp\left(\beta \right)-1\right)\#\; \end{array} \end{array}$$

where y is the natural logarithm of age-standardized rates, and T represents the calendar year.^[24]^ Positive EAPC values (EAPC > 0) indicated ascending temporal trends, while negative values (EAPC < 0) signified declining patterns. Statistical significance was established when the 95% CI of the EAPC excluded 0. Risk factor impact was evaluated through mortality and DALYs attributable to metabolic, dietary, and lifestyle factors. Spearman correlation assessed associations between age-standardized rates and SDI values across 204 countries in 2021, and relationships between temporal trends and SDI across regions from 1990 to 2021.

Frontier analysis was performed using a bootstrap data envelopment analysis approach to identify gaps between observed disease burden and optimal achievable performance at given SDI levels. One hundred bootstrap iterations were conducted with random sampling from all country–year observations (1990–2021). Within each iteration, observations were ordered by ascending SDI, and the frontier was defined as the cumulative minimum age-standardized rate at each SDI level. Final frontier values represented the mean across all bootstrap iterations. For each country–year observation, the performance gap was calculated as the difference between observed burden and the frontier value. For visualization, the frontier curve was smoothed using locally weighted scatterplot smoothing regression (bandwidth = 0.2).^[25]^

Future age-standardized rates through 2050 were projected using ARIMA modeling.^[18]^ For each metric-sex combination, candidate models with autoregressive (*p*), differencing (*d*), and moving average (*q*) parameters ranging from 0 to 5 (*p*, *q*) and 0 to 2 (*d*) were systematically evaluated using Akaike Information Criterion and Bayesian Information Criterion. Model adequacy was verified through Ljung–Box *Q* test for residual autocorrelation (*P* > .05) and out-of-sample validation using 2019 to 2021 data. Final selected models achieved mean absolute percentage error ranging from 0.27% to 1.10% (mean: 0.58%), indicating excellent predictive performance. All statistical analyses were performed using R language (version 4.4.2) (R Foundation for Statistical Computing, Vienna, Austria). Comprehensive data visualization was achieved through generation of figures and tables illustrating temporal trends, risk factor contributions, and future burden projections. Statistical significance was determined based on *P*-values, with *P* < .05 considered statistically significant.

## 3. Results

### 3.1. Burden and trends of CRC in the population aged 65 to 74 years

**Figure 1.**
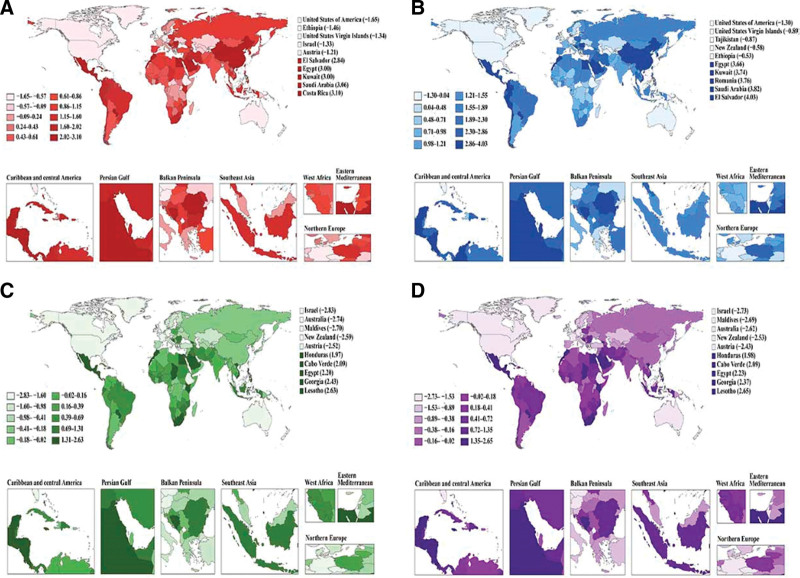
Geographic distribution of temporal trends in colorectal cancer burden among adults aged 65 to 74 years, 1990 to 2021. (A) Incidence. (B) Prevalence. (C) Mortality. (D) Disability-adjusted life years. Color gradient indicates estimated annual percentage change, with darker shades representing larger magnitude of change. Regional insets display Caribbean and Central America, Persian Gulf, Balkan Peninsula, Southeast Asia, West Africa, Eastern Mediterranean, and Northern Europe. Top 10 countries with highest estimated annual percentage change are listed for each metric. EAPC = estimated annual percentage change.

From 1990 to 2021, CRC incidence demonstrated modest but significant increase among individuals aged 65 to 74 years. ASIR rose from 125.03 (95% UI: 119.76–129.53) to 136.19 (95% UI: 125.63–147.15) per 100,000 person-years (EAPC 0.10%, 95% CI: 0.03%–0.16%; *P* = .006) (Table 1). Gender-stratified analyses revealed divergent trajectories: males experienced upward trends (EAPC 0.39%, 95% CI: 0.31–0.46%; *P* < .001), while females showed downward patterns (EAPC −0.36%, 95% CI: −0.43% to −0.29%; *P* < .001). Substantial geographic variation emerged across socioeconomic regions. Middle-income countries and Central Latin America exhibited steepest increases (EAPCs 1.68% and 2.17%), whereas high-income regions showed notable declines (EAPC −0.50%). Costa Rica had the highest EAPC (3.10%), followed by Saudi Arabia (3.06%), while the United States recorded the lowest (−1.65%) (Fig. 1A).

**Table 1:** Trends in age-standardized incidence, prevalence, mortality, and DALYs rates among colorectal cancer patients aged 65 to 74 years, 1990 to 2021.

Characteristics	Age-standardized incidence rate				Age-standardized prevalence rate				Age-standardized mortality rate				Age-standardized DALYs rate			
	1990 (95% UI)	2021 (95% UI)	EAPC (95% CI) 1990–2021	*P* value	1990 (95% UI)	2021 (95% UI)	EAPC (95% CI) 1990–2021	*P* value	1990 (95% UI)	2021 (95% UI)	EAPC (95% CI) 1990–2021	*P* value	1990 (95% UI)	2021 (95% UI)	EAPC (95% CI) 1990–2021	*P* value
Global	125.03 (119.76–129.53)	136.19 (125.63–147.15)	0.10 (0.03 to 0.16)	.006	596.30 (567.31–626.42)	758.84 (699.75–817.26)	0.69 (0.64 to 0.75)	<.001	73.41 (69.75–76.67)	58.48 (54.07–63.06)	−0.96 (−1.04 to −0.88)	<.001	1678.26 (1593.61–1749.49)	1354.97 (1255.11–1459.49)	−0.90 (−0.98 to −0.83)	<.001
Sex																
Female	106.89 (100.03–112.57)	102.71 (92.02–113.13)	−0.36 (−0.43 to −0.29)	<.001	524.75 (493.09–556.71)	584.52 (522.89–640.61)	0.21 (0.16 to 0.25)	<.001	62.58 (58.48–66.65)	44.45 (40.24–48.24)	−1.37 (−1.46 to −1.29)	<.001	1427.86 (1335.29–1519.37)	1027.42 (930.29–1116.84)	−1.32 (−1.40 to −1.24)	<.001
Male	146.82 (140.06–153.60)	173.49 (157.20–194.09)	0.39 (0.31 to 0.46)	<.001	682.04 (649.06–717.93)	952.94 (863.93–1061.88)	1.02 (0.95 to 1.10)	<.001	86.44 (81.31–91.53)	74.13 (67.24–82.52)	−0.70 (−0.78 to −0.61)	<.001	1978.23 (1865.14–2094.03)	1719.35 (1562.25–1916.35)	−0.64 (−0.72 to −0.57)	<.001
SDI quintiles																
High	233.71 (224.05–240.62)	210.69 (198.07–220.67)	−0.50 (−0.65 to −0.35)	<.001	1297.64 (1235.62–1355.58)	1341.05 (1259.69–1404.13)	0.02 (-0.14 to 0.19)	.765	106.45 (102.50–109.25)	68.03 (64.27–70.87)	−1.67 (−1.77 to −1.57)	<.001	2465.06 (2373.57–2538.51)	1609.63 (1517.64–1686.53)	−1.58 (−1.67 to −1.49)	<.001
High-middle	135.42 (127.83–141.85)	183.59 (164.21–205.78)	0.90 (0.81 to 0.98)	<.001	574.02 (539.55–610.40)	1019.59 (909.44–1144.52)	1.93 (1.82 to 2.03)	<.001	90.22 (85.30–94.85)	77.52 (70.10–85.87)	−0.69 (−0.81 to −0.57)	<.001	2051.07 (1940.12–2156.81)	1796.71 (1626.64–1992.99)	−0.62 (−0.74 to −0.51)	<.001
Middle	63.95 (57.08–71.00)	105.69 (91.55–120.97)	1.68 (1.61 to 1.75)	<.001	223.75 (200.76–248.95)	524.35 (451.86–599.53)	2.97 (2.87 to 3.07)	<.001	52.00 (46.51–57.76)	52.82 (46.47–59.71)	0.01 (−0.03 to 0.05)	.507	1172.65 (1048.30–1301.19)	1212.42 (1065.46–1368.36)	0.07 (0.03 to 0.11)	.001
Low-middle	31.87 (27.54–36.25)	43.16 (39.34–47.22)	0.90 (0.84 to 0.97)	<.001	92.06 (80.73–104.08)	152.19 (138.91–166.74)	1.59 (1.53 to 1.65)	<.001	29.62 (25.50–33.79)	33.95 (31.06–37.24)	0.36 (0.30 to 0.43)	<.001	669.89 (577.03–764.52)	769.77 (703.53–843.42)	0.37 (0.31 to 0.44)	<.001
Low	40.91 (32.28–46.84)	37.97 (33.76–42.60)	−0.30 (−0.40 to −0.21)	<.001	102.70 (83.33–117.25)	113.01 (100.94–126.13)	0.27 (0.15 to 0.38)	<.001	40.09 (31.67–45.94)	33.99 (30.17–38.12)	−0.58 (−0.65 to −0.51)	<.001	903.47 (714.64–1033.82)	766.65 (680.91–861.37)	−0.59 (−0.67 to −0.52)	<.001
GBD regions																
Andean Latin America	51.52 (42.49–61.70)	79.40 (60.72–102.30)	1.45 (1.35 to 1.56)	<.001	198.36 (166.07–237.92)	430.36 (327.66–554.09)	2.70 (2.57 to 2.84)	<.001	45.38 (37.39–53.86)	49.67 (38.54–63.76)	0.33 (0.22 to 0.43)	<.001	1020.18 (839.89–1210.87)	1130.94 (875.65–1449.47)	0.36 (0.26 to 0.46)	<.001
Australasia	276.83 (247.43–306.09)	214.20 (181.99–249.18)	−1.11 (−1.43 to −0.78)	<.001	1524.10 (1358.09–1689.21)	1421.79 (1216.87–1646.57)	−0.42 (−0.81 to −0.03)	.037	127.06 (114.38–140.27)	61.99 (53.71–70.80)	−2.72 (−2.91 to −2.52)	<.001	2940.10 (2650.78–3246.04)	1479.58 (1275.28–1705.60)	−2.61 (−2.80 to −2.42)	<.001
Caribbean	132.36 (121.01–145.38)	196.61 (167.56–226.70)	1.46 (1.39 to 1.53)	<.001	565.51 (511.18–624.91)	1021.86 (866.89–1175.04)	2.20 (2.08 to 2.32)	<.001	67.61 (62.10–73.56)	71.21 (61.78–81.60)	0.31 (0.26 to 0.36)	<.001	1542.73 (1417.04–1683.18)	1661.61 (1440.10–1905.47)	0.38 (0.33 to 0.44)	<.001
Central Asia	72.29 (66.56–78.12)	65.97 (58.64–72.84)	−0.05 (−0.21 to 0.11)	.536	247.67 (228.39–269.43)	259.78 (232.37–287.19)	0.46 (0.22 to 0.71)	.001	59.01 (54.47–63.59)	46.97 (41.97–51.81)	−0.56 (−0.65 to −0.47)	<.001	1342.45 (1238.45–1448.26)	1063.54 (951.19–1173.11)	−0.58 (−0.68 to −0.48)	<.001
Central Europe	157.25 (149.02–165.50)	232.58 (213.64–252.95)	1.15 (0.98 to 1.33)	<.001	595.19 (558.16–634.49)	1128.82 (1026.69–1232.52)	2.12 (1.91 to 2.32)	<.001	116.60 (110.84–122.14)	124.41 (114.61–134.03)	0.01 (−0.13 to 0.15)	.886	2647.45 (2519.13–2775.04)	2851.07 (2627.07–3072.81)	0.06 (−0.07 to 0.19)	.373
Central Latin America	49.52 (46.89–52.15)	99.95 (87.68–112.63)	2.17 (2.10 to 2.24)	<.001	180.16 (168.81–191.58)	460.89 (404.14–518.78)	3.03 (2.95 to 3.11)	<.001	33.99 (32.23–35.82)	45.72 (40.39–51.14)	0.90 (0.83 to 0.97)	<.001	767.37 (728.51–808.46)	1053.47 (932.57–1180.73)	0.96 (0.89 to 1.04)	<.001
Central Sub-Saharan Africa	40.79 (31.12–52.06)	42.73 (31.87–56.43)	0.20 (0.04 to 0.36)	.018	102.79 (79.65–130.83)	123.29 (93.33–161.46)	0.64 (0.43 to 0.86)	<.001	40.09 (30.59–51.22)	39.30 (29.27–52.48)	−0.00 (−0.13 to 0.13)	.996	903.42 (690.48–1153.30)	888.47 (663.10–1187.01)	0.00 (−0.13 to 0.14)	.966
East Asia	89.70 (76.97–102.56)	161.74 (130.85–195.09)	1.99 (1.91 to 2.06)	<.001	329.39 (285.42–376.70)	906.57 (740.34–1088.38)	3.53 (3.40 to 3.65)	<.001	69.69 (59.63–79.78)	63.93 (51.81–77.24)	−0.37 (−0.47 to −0.27)	<.001	1570.80 (1344.09–1798.80)	1481.43 (1199.29–1786.91)	−0.27 (−0.36 to −0.18)	<.001
Eastern Europe	150.74 (142.75–158.62)	193.44 (177.42–210.25)	0.62 (0.52 to 0.72)	<.001	601.16 (565.00–642.94)	919.93 (846.43–996.76)	1.34 (1.17 to 1.52)	<.001	105.00 (99.80–110.02)	102.77 (93.75–112.21)	−0.34 (−0.49 to −0.20)	<.001	2393.64 (2271.95–2508.50)	2362.81 (2159.37–2580.90)	−0.32 (−0.46 to −0.18)	<.001
Eastern Sub-Saharan Africa	63.52 (49.69–73.93)	57.08 (49.47–66.06)	−0.45 (−0.55 to −0.35)	<.001	153.65 (124.17–175.94)	167.18 (145.64–193.12)	0.20 (0.07 to 0.32)	.003	62.83 (49.11–73.21)	51.85 (44.97–60.08)	−0.71 (−0.80 to −0.63)	<.001	1413.39 (1106.25–1645.10)	1167.69 (1013.34–1354.29)	−0.72 (−0.81 to −0.64)	<.001
High-income Asia Pacific	202.18 (189.79–213.27)	249.07 (223.29–271.79)	0.44 (0.29 to 0.60)	<.001	1213.12 (1135.15–1295.85)	1661.28 (1497.47–1807.48)	0.81 (0.66 to 0.97)	<.001	83.86 (79.84–87.30)	69.48 (63.58–73.59)	−0.79 (−0.89 to −0.70)	<.001	1952.18 (1856.81–2035.29)	1661.60 (1523.74–1766.89)	−0.70 (−0.80 to −0.61)	<.001
High-income North America	266.45 (252.09–277.01)	182.24 (170.84–191.63)	−1.53 (−1.72 to −1.33)	<.001	1568.49 (1484.86–1651.51)	1183.20 (1110.26–1241.96)	−1.15 (−1.36 to −0.95)	<.001	104.56 (99.60–108.61)	55.13 (51.71–57.58)	−2.32 (−2.44 to −2.21)	<.001	2442.21 (2329.18–2546.13)	1316.81 (1240.05–1383.21)	−2.24 (−2.35 to −2.13)	<.001
North Africa and Middle East	49.32 (41.95–57.09)	73.83 (62.75–85.77)	1.40 (1.29 to 1.51)	<.001	191.04 (166.07–219.16)	397.90 (338.69–464.23)	2.49 (2.39 to 2.59)	<.001	42.15 (35.81–49.03)	43.77 (37.39–50.57)	0.17 (0.08 to 0.26)	.001	954.06 (808.51–1108.28)	1002.29 (854.45–1160.95)	0.21 (0.11 to 0.30)	<.001
Oceania	32.05 (26.58–38.40)	31.68 (26.70–37.72)	−0.11 (−0.18 to −0.03)	.005	95.94 (80.97–112.55)	105.14 (89.79–122.83)	0.19 (0.09 to 0.28)	<.001	28.95 (23.91–34.77)	26.41 (22.04–31.53)	−0.33 (−0.40 to −0.26)	<.001	656.08 (542.34–787.03)	598.58 (499.86–715.57)	−0.33 (−0.40 to −0.26)	<.001
South Asia	24.75 (21.00–28.49)	30.29 (26.81–33.98)	0.42 (0.26 to 0.57)	<.001	67.28 (58.11–77.47)	100.47 (89.49–112.71)	1.08 (0.92 to 1.24)	<.001	23.55 (19.95–27.16)	24.68 (21.85–27.76)	−0.07 (−0.21 to 0.07)	.300	534.27 (452.85–615.94)	559.74 (495.79–629.89)	−0.07 (−0.21 to 0.07)	.331
Southeast Asia	58.34 (49.65–67.12)	94.03 (80.01–108.03)	1.54 (1.46 to 1.61)	<.001	180.83 (155.30–206.13)	376.73 (320.98–431.53)	2.40 (2.34 to 2.47)	<.001	52.24 (44.30–60.14)	65.53 (56.29–74.78)	0.73 (0.65 to 0.81)	<.001	1179.90 (998.43–1356.08)	1490.94 (1278.88–1705.69)	0.75 (0.67 to 0.82)	<.001
Southern Latin America	131.38 (115.99–148.11)	153.28 (132.65–175.19)	0.64 (0.47 to 0.80)	<.001	481.73 (422.02–542.76)	708.50 (613.85–806.96)	1.46 (1.29 to 1.63)	<.001	102.48 (91.20–115.00)	90.26 (79.35–102.74)	−0.24 (−0.38 to −0.09)	.002	2307.29 (2047.23–2597.66)	2058.47 (1808.34–2345.46)	−0.20 (−0.34 to −0.06)	.008
Southern Sub-Saharan Africa	42.86 (36.71–54.41)	66.75 (58.52–74.94)	1.60 (1.32 to 1.87)	<.001	132.89 (113.94–168.50)	217.22 (191.74–243.75)	1.74 (1.60 to 1.87)	<.001	38.20 (32.73–48.50)	54.29 (47.74–61.00)	1.31 (0.99 to 1.64)	<.001	863.33 (738.06–1100.44)	1232.41 (1083.06–1380.76)	1.33 (1.00 to 1.65)	<.001
Tropical Latin America	57.44 (53.47–61.63)	90.99 (82.60–99.18)	1.40 (1.25 to 1.55)	<.001	195.49 (181.91–210.34)	396.98 (360.51–433.89)	2.25 (2.11 to 2.39)	<.001	46.78 (43.58–50.01)	57.29 (52.43–62.11)	0.62 (0.50 to 0.73)	<.001	1052.53 (982.34–1125.41)	1306.12 (1193.87–1414.30)	0.65 (0.54 to 0.76)	<.001
Western Europe	228.24 (217.45–238.26)	221.37 (204.52–236.32)	−0.19 (−0.38 to −0.01)	.042	1217.50 (1152.04–1282.80)	1423.30 (1319.39–1516.81)	0.50 (0.28 to 0.73)	<.001	110.80 (106.15–114.82)	69.69 (64.86–73.95)	−1.65 (−1.74 to −1.56)	<.001	2553.67 (2445.54–2650.36)	1652.14 (1536.42–1758.26)	−1.54 (−1.63 to −1.46)	<.001
Western Sub-Saharan Africa	27.68 (23.61–32.26)	32.56 (26.78–38.26)	0.68 (0.61 to 0.75)	<.001	72.58 (62.41–84.36)	94.08 (78.04–110.34)	1.00 (0.90 to 1.11)	<.001	26.81 (22.92–31.30)	29.25 (24.11–34.24)	0.45 (0.38 to 0.51)	<.001	600.44 (513.37–701.17)	656.73 (540.71–770.33)	0.45 (0.38 to 0.51)	<.001

CI = confidence interval, EAPC = estimated annual percentage change, GBD = Global Burden of Diseases, SDI = socio-demographic index, UI = uncertainty interval.

Between 1990 and 2021, CRC prevalence exhibited pronounced upward trends globally. ASPR increased from 596.30 (95% UI: 567.31–626.42) to 758.84 (95% UI: 699.75–817.26) per 100,000 population (EAPC 0.69%, 95% CI: 0.64–0.75%; *P* < .001). Males demonstrated greater prevalence escalation (EAPC 1.02%) than females (EAPC 0.21%). Geographic patterns varied substantially, with middle-income territories leading prevalence expansion (EAPC 2.97%). East Asia achieved the highest regional growth (EAPC 3.53%), followed by Central Latin America (EAPC 3.03%). High-income North America was the sole region showing prevalence reduction (EAPC − 1.15%). Country-level analysis identified El Salvador, Saudi Arabia, and Romania as prevalence hotspots, while the United States, United States Virgin Islands, and Tajikistan achieved significant reductions (Fig. 1B).

Over the study period (1990–2021), mortality outcomes revealed encouraging global improvements. ASMR decreased substantially from 73.41 (95% UI: 69.75–76.67) in 1990 to 58.48 (95% UI: 54.07–63.06) per 100,000 person-years in 2021 (EAPC − 0.96%, 95% CI: −1.04% to −0.88%; *P* < .001). Sex-specific analysis confirmed persistent gender disparities: females achieved superior mortality reduction (EAPC −1.37%) compared to males (EAPC −0.70%), despite males maintaining higher absolute rates (74.13 vs 44.45 per 100,000 person-years in 2021). Regional analysis showed significant variation across socioeconomic levels. High SDI countries attained substantial mortality improvements (EAPC −1.67%), with Australasia (EAPC −2.72%) and high-income North America (EAPC −2.32%) demonstrating exceptional progress. Conversely, resource-limited regions experienced worsening trends, particularly Southern Sub-Saharan Africa (EAPC 1.31%) and Central Latin America (EAPC 0.90%). Israel achieved the lowest EAPC (−2.83%), followed by Australia (−2.74%), while Lesotho had the highest EAPC (2.63%) (Fig. 1C).

From 1990 to 2021, DALYs demonstrated favorable trends globally. ASDR declined markedly from 1678.26 (95% UI: 1593.61–1749.49) to 1354.97 (95% UI: 1255.11–1459.49) per 100,000 population (EAPC − 0.90%, 95% CI: −0.98% to −0.83%; *P* < .001). Gender-specific burden patterns showed females experiencing greater burden alleviation (EAPC −1.32%) relative to males (EAPC −0.64%). Geographic burden distribution varied significantly across regions. Developed regions led burden reduction efforts, with Australasia (EAPC −2.61%, 95% CI: −2.80% to −2.42%; *P* < .001) and high-income North America (EAPC −2.24%, 95% CI: −2.35% to −2.13%; *P* < .001) achieving remarkable burden mitigation. In contrast, developing regions experienced burden intensification, particularly Southern Sub-Saharan Africa (EAPC 1.33%) and Central Latin America (EAPC 0.96%). National-level performance demonstrated considerable heterogeneity, with Israel achieving exceptional burden reduction (EAPC −2.73%) and the Maldives showing remarkable progress (EAPC −2.69%), while concerning burden escalations occurred in Lesotho (EAPC 2.65%) and Georgia (EAPC 2.37%) (Fig. 1D).

### 3.2. Relationship between SDI and the ASIR and ASMR

**Figure 2.**
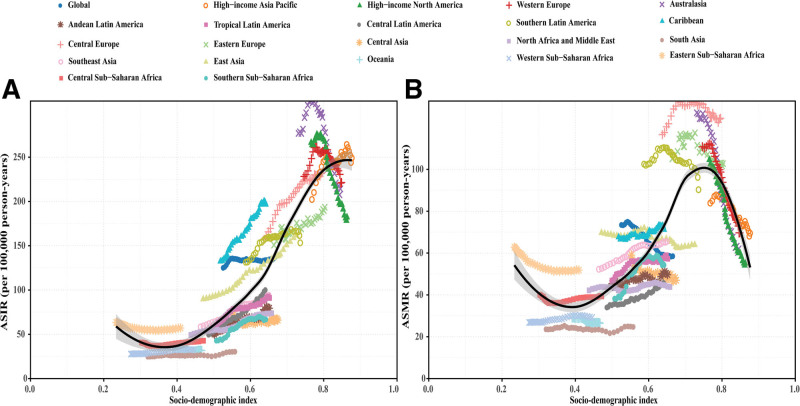
Association between socio-demographic index and colorectal cancer burden among individuals aged 65 to 74 years globally and across 21 Global Burden of Disease regions, 1990 to 2021. (A) ASIR. (B) ASMR. Each data point represents 1 year for each region, with different colors distinguishing regions. Black curve represents the overall temporal trend across all regions; gray shaded area indicates 95% confidence interval. Deviation from the curve reflects regional variation in disease burden beyond socioeconomic factors. ASIR = age-standardized incidence rate, ASMR = age-standardized mortality rate.

**Figure 3.**
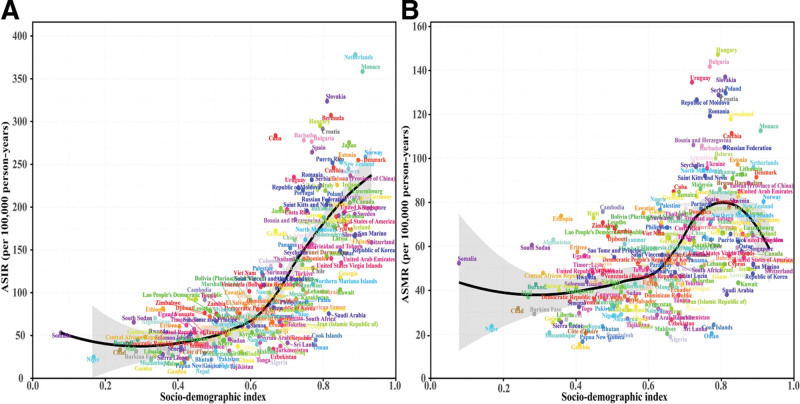
Colorectal cancer burden among individuals aged 65 to 74 years across 204 countries and territories by socio-demographic index in 2021. (A) ASIR. (B) ASMR. Each labeled point represents 1 country or territory. Black curve represents expected relationship between socio-demographic index and disease burden; gray shaded area indicates 95% confidence interval. Points above the curve indicate higher-than-expected burden; points below indicate lower-than-expected burden. ASIR = age-standardized incidence rate, ASMR = age-standardized mortality rate.

CRC burden demonstrated positive association with socioeconomic development. ASIRs were lowest in low-SDI regions (<50 per 100,000 person-years) and increased with development level, exceeding 200 per 100,000 person-years in high SDI regions (Spearman *R* = 0.898, *P* < .001) (Fig. 2A). ASMRs increased sharply in middle SDI regions (60–90 per 100,000 person-years), then decreased in high SDI regions (approximately 90 to 50 per 100,000 person-years) (Spearman *R* = 0.749, *P* < .001) (Fig. 2B). At national level, ASIRs exhibited upward trends with increasing SDI, consistent with regional patterns (Spearman *R* = 0.817, *P* < .001). Similarly, ASMRs showed downward trends in high SDI countries (Spearman *R* = 0.567, *P* < .001) (Fig. 3).

### 3.3. Frontier analysis

**Figure 4.**
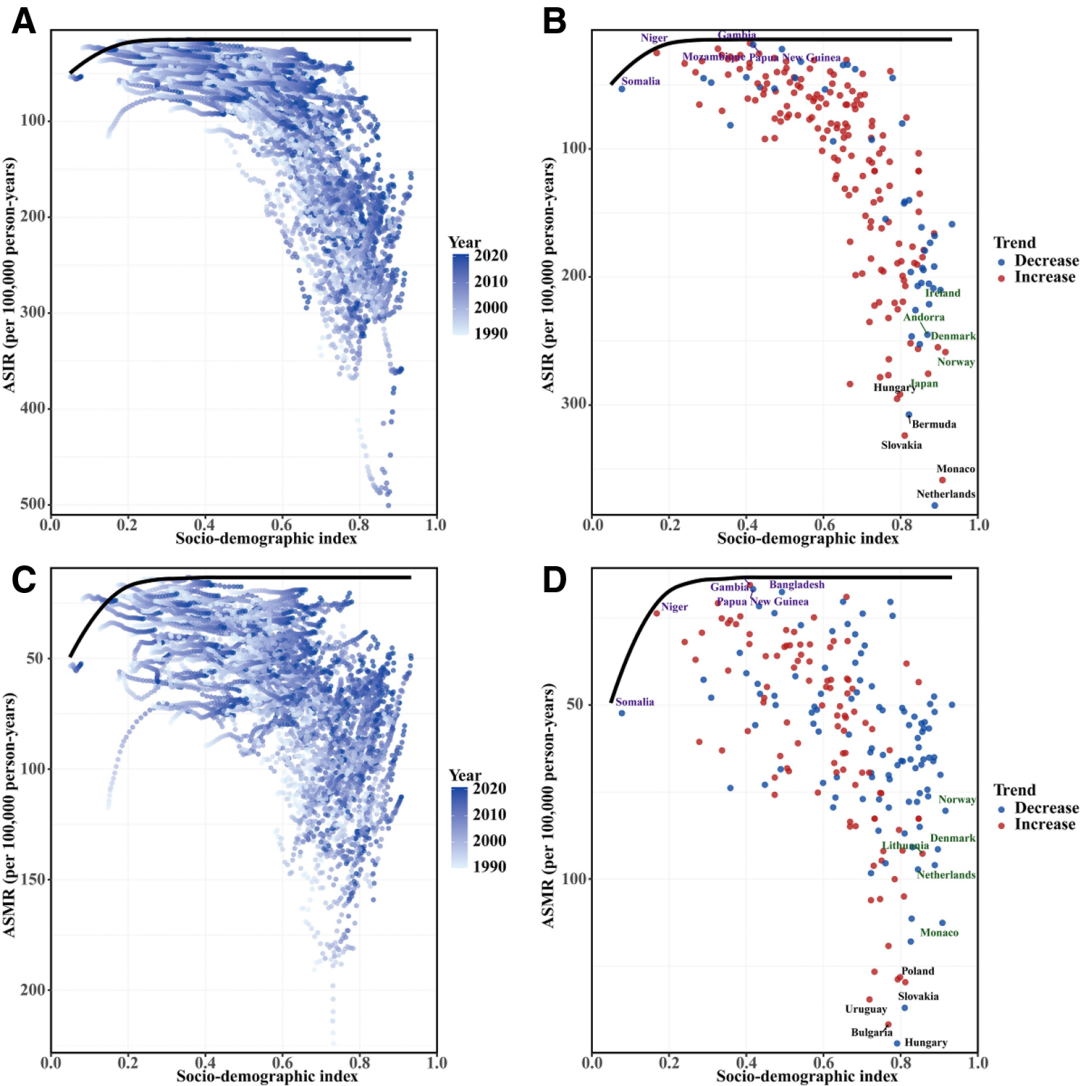
Frontier analysis of improvement gaps in colorectal cancer burden among individuals aged 65 to 74 years using country-level data from 1990 to 2021. (A, C) ASIR and ASMR for all years (1990–2021). (B, D) Corresponding rates for 2021 only, colored by temporal trend direction. Solid black curve represents frontier (optimal performance at each socio-demographic index level). Selected countries are labeled: black text identifies 5 countries with largest gaps from frontier; purple text identifies 5 low-SDI countries (SDI < 0.5) with minimal gaps; green text identifies 5 high SDI countries (SDI > 0.85) with large gaps. Red dots indicate increasing rates (1990–2021); blue dots indicate decreasing rates. ASIR = age-standardized incidence rate, ASMR = age-standardized mortality rate, SDI = socio-demographic index.

Frontier analyses revealed substantial improvement potential. For ASIRs, the Netherlands, Monaco, Slovakia, Bermuda, and Hungary exhibited widest disparities between observed and frontier-predicted rates. Countries with SDI below 0.5 (including Somalia, Niger, Gambia, Papua New Guinea, and Mozambique) displayed lowest absolute rates with minimal deviations. Several high-development nations (SDI > 0.85) achieved superior outcomes, with Ireland, Andorra, Denmark, Norway, and Japan demonstrating rates below frontier projections (Fig. 4A and B). Mortality analysis identified different suboptimal performance patterns: Hungary, Bulgaria, Uruguay, Slovakia, and Poland exhibited most pronounced discrepancies between actual and expected death rates (Fig. 4C and D).

### 3.4. Disease burden and trends attributable to main risk factors

**Figure 5.**
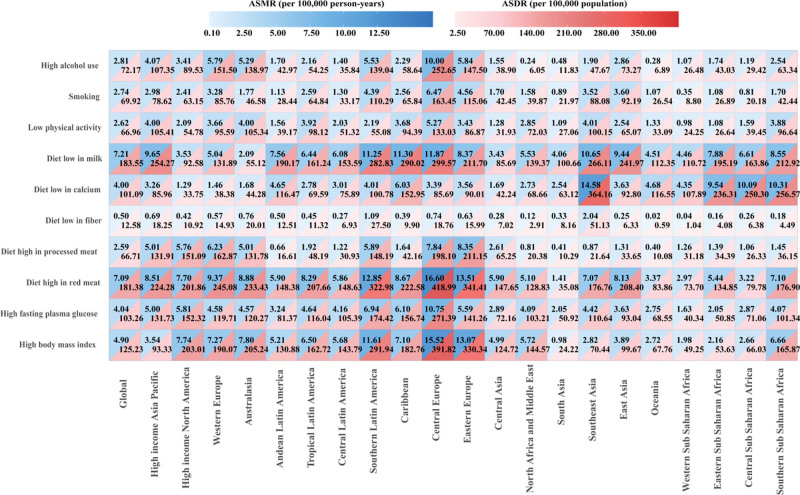
Risk factor contributions to colorectal cancer burden among individuals aged 65 to 74 years in 2021. Heat map displays ASMR (left panel, blue gradient) and ASDR (right panel, red gradient) attributable to major risk factors globally and across 21 Global Burden of Disease regions. Rows represent 10 risk factors categorized as dietary, behavioral, and metabolic. Cell values indicate burden magnitude; darker colors indicate higher attributable burden. ASMR = age-standardized mortality rate, ASDR = age-standardized disability-adjusted life years rate.

From 1990 to 2021, CRC ASMR and ASDR attributable to major modifiable risk factors among individuals aged 65 to 74 years showed substantial global and regional variations. Low milk consumption was the leading contributor globally, with ASMR and ASDR of 7.21 per 100,000 person-years and 183.55 per 100,000 population, respectively, in 2021. Both rates declined globally (ASMR EAPC −0.71%, 95% CI: −0.80% to −0.62%; ASDR EAPC −0.65%, 95% CI: −0.74% to −0.56%; both *P* < .001), with slight increases in low-middle SDI regions. High red meat consumption ranked second (ASMR 7.09 per 100,000 person-years; ASDR 181.38 per 100,000 population), with global declines in both rates (ASMR EAPC −1.03%, 95% CI: −1.10% to −0.96%; ASDR EAPC −0.97%, 95% CI: −1.03% to −0.90%; both *P* < .001) but increases in middle and low-middle SDI regions. High BMI ranked 3rd (ASMR 4.90 per 100,000 person-years; ASDR 125.23 per 100,000 population), decreasing in high SDI regions but increasing substantially in low-middle SDI regions (ASMR EAPC 2.76%, 95% CI: 2.69–2.83%; ASDR EAPC 2.78%, 95% CI: 2.71–2.85%; both *P* < .001) (Table S1, Supplemental Digital Content, https://links.lww.com/MD/R310). Geospatial analysis identified Central Europe as a hotspot, showing the highest ASMR and ASDR across most risk factors in 2021 (Fig. 5).

### 3.5. Prediction and changing trends of disease burden from 2022 to 2050

**Figure 6.**
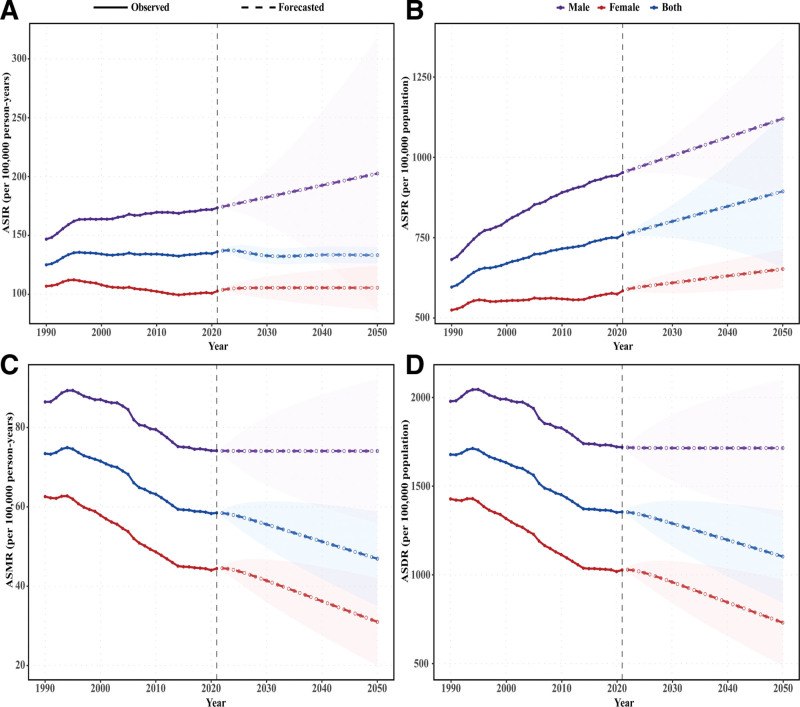
Historical trends and projected colorectal cancer burden among individuals aged 65 to 74 years by sex, 1990 to 2050. (A) ASIR. (B) ASPR. (C) ASMR. (D) ASDR. Solid lines represent observed rates (1990–2021); dashed lines with circular markers represent forecasted rates (2022–2050) generated using autoregressive integrated moving average modeling. Purple lines indicate males, red lines indicate females, and blue lines indicate both sexes. Shaded areas indicate 95% confidence intervals for forecasts. Vertical dashed line demarcates 2021. ASIR = age-standardized incidence rate, ASPR = age-standardized prevalence rate, ASMR = age-standardized mortality rate, ASDR = age-standardized disability-adjusted life years rate.

From 2022 to 2050, the global ASIR of CRC among individuals aged 65 to 74 years is projected to decline slightly, from 137.11 in 2022 to 133.24 per 100,000 person-years in 2050 (EAPC −0.07%, 95% CI: −0.12% to −0.03%; *P* = .001). In contrast, ASPR is expected to rise steadily, increasing from 763.51 to 894.15 per 100,000 population (EAPC 0.57%, 95% CI: 0.56–0.57%; *P* < .001). ASMR is predicted to decrease from 58.43 to 46.92 per 100,000 person-years (EAPC −0.80%, 95% CI: −0.82% to −0.78%; *P* < .001), accompanied by a decline in ASDR from 1353.26 to 1103.45 per 100,000 population (EAPC −0.74%, 95% CI: −0.76% to −0.73%; *P* < .001). Sex disparities remain evident. By 2050, males are estimated to have markedly higher ASIR (202.62 vs 105.57 per 100,000 person-years) and ASPR (1120.33 vs 652.50 per 100,000 population) compared with females. Mortality and DALYs burdens are also greater in men, with ASMR at 74.06 vs 31.02 per 100,000 person-years and ASDR at 1714.72 vs 730.83 per 100,000 population (Fig. 6). Regional patterns reveal substantial variation in projected disease burden across global territories. High-income North America is projected to experience the largest increase in ASIR, rising from 186.11 to 294.45 per 100,000 person-years (EAPC 1.64%). Central Latin America and Tropical Latin America demonstrate moderate increases in incidence (EAPCs 1.33% and 1.02%, respectively) with corresponding disability burden elevations (EAPCs 0.78% and 0.17%, respectively). In contrast, Western Europe exhibits declining trends across mortality and disability metrics, with ASMR decreasing from 68.94 to 33.39 per 100,000 person-years (EAPC −2.53%) and ASDR falling from 1634.31 to 852.41 per 100,000 population (EAPC −2.28%). East Asia demonstrates increasing incidence and prevalence trends (EAPCs 1.19% and 1.91%, respectively) while maintaining stable mortality and disability rates (Table S2, Supplemental Digital Content, https://links.lww.com/MD/R310).

## 4. Discussion

CRC represents a substantial global health challenge among older adults.^[26]^ This study suggests distinctive epidemiological patterns in the 65 to 74 years demographic that distinguish it from broader age cohorts. The modest global incidence increases alongside substantial mortality reductions may indicate enhanced therapeutic effectiveness specific to this population. Our findings extend beyond previous GBD studies by demonstrating age-specific trends that warrant targeted interventions.^[27,28]^ While earlier research emphasized early-onset disease as the primary driver of escalating global incidence, our age-specific findings reveal that young-older adults appear to experience a different risk profile requiring specialized approaches.^[29]^ The demographic specificity may reflect both preserved functional capacity enabling better treatment tolerance and optimal screening program benefits in this population.^[30]^ These patterns underscore the necessity for age-stratified approaches, as risk factor predominance and survival outcomes vary considerably across age strata.^[31]^

The pronounced sex-based epidemiological divergence (with males showing ascending incidence trends while females demonstrate declining patterns) may reflect complex biological and behavioral mechanisms. Beyond differential carcinogenic exposures including tobacco use, alcohol consumption, and dietary patterns,^[32]^ recent molecular investigations suggest fundamental sex-specific differences in CRC biology. Histone demethylase KDM5D upregulation appears to drive distinct tumor characteristics between sexes,^[33]^ while sex-specific survival patterns emerge in early-onset disease.^[34]^ These biological differences likely interact with hormonal factors and metabolic pathways that change distinctly in aging males versus females. The observed patterns suggest that post-menopausal hormonal changes in women may provide continued protective effects, while age-related testosterone decline in men coincides with increased susceptibility to inflammatory and metabolic risk factors.

Regional burden disparities may reflect healthcare system maturation and prevention infrastructure effectiveness. High-income territories achieved substantial mortality improvements despite elevated incidence rates through successful integration of population-based screening with advanced therapeutics.^[9]^ This contrasts with middle-income countries experiencing epidemiological transition, where urbanization and dietary westernization occur without corresponding healthcare infrastructure development.^[5]^ Large randomized trials have confirmed colonoscopy screening effectiveness specifically in older adult populations.^[10]^ The temporal alignment between screening program implementation and mortality reductions suggests causality, though birth cohort effects and concurrent therapeutic advances also contribute to observed trends.

Countries with advanced socio-demographic indices showing suboptimal performance suggest inefficient resource allocation rather than biological constraints. The frontier analysis reveals that theoretical optimal rates may be achievable through systematic healthcare delivery improvements. However, biological limits likely prevent mortality rates from approaching 0, and the analysis should account for competing mortality risks in this age group. Nations achieving superior performance appear to demonstrate effective integration of screening programs with age-appropriate clinical pathways, indicating that technological capacity requires systematic implementation strategies to achieve maximum benefit.

The predominance of dietary determinants provides actionable intervention targets with considerable population health impact potential. Insufficient milk consumption emerged as the primary attributable risk factor, followed by excessive red meat intake, highlighting the critical importance of adequate calcium consumption and processed meat limitation. Large-scale prospective studies have confirmed the protective effects of milk consumption against CRC development, with substantial evidence from Mendelian randomization studies reinforcing causal evidence for dairy products’ protective role.^[35]^ Comprehensive meta-analyses continue to demonstrate red and processed meat consumption’s association with increased CRC risk, revealing significant dose–response relationships.^[36,37]^ These dietary effects may be amplified in older adults due to age-related changes in intestinal barrier function and immune response capacity. The prominence of high BMI as a major risk factor emphasizes the expanding global burden of metabolic dysfunction in aging populations, with recent umbrella reviews confirming obesity’s 25% to 57% elevation in CRC risk.^[38,39]^ The metabolic dysfunction underlying obesity creates pro-inflammatory conditions particularly relevant to aging populations. Temporal increases in elevated fasting glucose reflect expanding diabetes prevalence, which significantly increases CRC risk through multiple pathways.^[40]^ Type 2 diabetes affects both colon and rectal cancer development through hyperinsulinemia, chronic inflammation, and altered gut microbiome composition.^[41,42]^ The convergence of metabolic dysfunction with age-related immune system changes may explain the particularly strong associations observed in this demographic. Additionally, the geographic concentration of risk factor burden in specific regions emphasizes the urgent need for culturally sensitive, region-specific interventions addressing local dietary traditions, economic constraints, and healthcare accessibility challenges.^[43]^ These findings support region-specific interventions for middle-income countries experiencing rapid CRC burden increases among young-older adults aged 65 to 74 years. Cost-effective fecal immunochemical testing-based screening programs should prioritize Central Latin America and East Asia, where incidence rises fastest at 2.17% and 1.99% annually, respectively. Dietary campaigns should be tailored to promote dairy consumption in low-intake regions while reducing processed meat intake in high-burden areas. Additionally, gender-sensitive strategies are needed to address divergent trends, with males showing ascending patterns (0.39% annually) and females exhibiting declining trends (−0.36% annually). These targeted interventions can leverage existing healthcare infrastructure while addressing predominant modifiable risk factors, potentially achieving substantial health gains at modest costs in resource-limited settings.

Our projections through 2050 indicate persistent regional heterogeneity with continued challenges in middle-income regions while developed countries demonstrate improving mortality trajectories. These patterns reflect differential healthcare system evolution and prevention program implementation success. Recent comprehensive analyses of evolving CRC landscapes projected continued global burden increases, with deaths potentially reaching 2.18 million by 2050, reinforcing the critical importance of age-specific prevention strategies identified in our analysis.^[28]^ Understanding these projections requires considering birth cohort effects, as individuals reaching 65 to 74 years by 2050 experienced different early-life exposures and healthcare throughout their life-course. Cost-effectiveness analyses demonstrate that targeted interventions in this age group may provide favorable economic returns,^[44]^ supporting continued investment in age-specific prevention strategies.

This study acknowledges several methodological limitations that warrant careful consideration in result interpretation. While the GBD methodology provides comprehensive global coverage, variations in data quality and reporting methodologies, particularly in low-income and middle-income countries, may introduce inaccuracies in CRC burden estimates among young older adults.^[45]^ These discrepancies could lead to under- or overestimation of disease burden, especially in regions with weaker health data systems and limited cancer registry infrastructure. Our risk factor analysis relied on modeled estimates and existing literature, potentially overlooking emerging or poorly characterized environmental and genetic risk factors specific to the 65 to 74 age demographic. Although the ARIMA model employed for future projections demonstrates robustness,^[46]^ it may not fully capture future discontinuities that could substantially alter CRC trends. Specifically, these projections assume trend continuation and may not account for paradigm shifts in 3 key areas. First, emerging screening technologies such as blood-based biomarkers and AI-enhanced colonoscopy may improve early detection beyond historical rates. Second, healthcare systems may adapt through upper age limit adjustments and senior- tailored screening programs. Third, dietary patterns may shift due to Western diet globalization or increasing plant-based adoption among aging populations. Consequently, these projections represent plausible trend-based scenarios rather than deterministic predictions. These inform proactive policy planning for this high-burden yet intervention-responsive demographic while acknowledging substantial forecasting uncertainty. Finally, the absence of histopathological data precluded subtype-specific analysis, limiting understanding of whether observed trends reflect changes in proximal versus distal cancers or different molecular subtypes that may respond differently to interventions.

These age-specific findings support developing targeted screening protocols that optimize the balance between benefits and potential harms in the 65 to 74 demographic. The enhanced therapeutic responsiveness observed in this group, combined with modifiable dietary risk factors, suggests that intensive prevention programs could yield substantial returns. However, interventions must account for the biological sex differences and regional healthcare infrastructure variations identified in this analysis. Future research should focus on developing personalized prevention strategies that consider individual risk profiles, biological sex, and regional healthcare capacity to optimize population-level outcomes while maintaining cost-effectiveness.

## 5. Conclusion

Young-older adults aged 65 to 74 years exhibit CRC epidemiological patterns characterized by modest global incidence increases alongside substantial mortality reductions, with pronounced sex disparities and regional inequalities. Males demonstrate ascending incidence trends while females exhibit declining patterns, necessitating sex-specific prevention strategies. Modifiable dietary factors, particularly insufficient milk consumption and excessive red meat intake, constitute primary intervention targets. Projected trends through 2050 emphasize the need for age-appropriate, sex-tailored prevention frameworks integrating optimized screening protocols, dietary modification programs, and comprehensive metabolic health management.

## Acknowledgments

We highly appreciate the works by the Global Burden of Disease Study 2021 collaborators.

## Author contributions

**Conceptualization:** Cailu Shen, Caier Cai, Yong Mao.

**Data curation:** Cailu Shen, Caier Cai.

**Formal analysis:** Cailu Shen, Caier Cai, Ying Zhang.

**Methodology:** Cailu Shen, Ying Zhang, Hejia Xu.

**Project administration:** Cailu Shen, Yong Mao.

**Software:** You Wu, Tong Xu.

**Supervision:** Tong Xu

**Validation:** Hejia Xu, You Wu.

**Writing – original draft:** Cailu Shen, Caier Cai, Ying Zhang.

**Writing – review & editing:** Cailu Shen, Caier Cai, Ying Zhang, Hejia Xu, You Wu, Tong Xu, Yong Mao.

## Supplementary Material


