# Supplementary material for: Global burden of colorectal cancer and associated risk factors in young-older adults: Trends from 1990 to 2021 with projections to 2050
Source: Medicine (Baltimore). 2026 Feb 6;105(6):e47448. doi: 10.1097/MD.0000000000047448 (PMC12885688; doi:10.1097/MD.0000000000047448)
Supplement: Supplementary file 1 [file medi-105-e47448-s001.pdf]

Table S1

Major risk factors contributing to age-standardized mortality and DALYs rates of colorectal cancer, and the corresponding EAPC, among individuals aged 65–74 years, 1990–2021.

Risk factors by SDI	Age-standardized mortality rate				Age-standardized DALYs rate			
	1990 (95% UI)	2021 (95% UI)	EAPC (95% CI) 1990–2021	*P* value	1990 (95% UI)	2021 (95% UI)	EAPC (95% CI) 1990–2021	*P* value
High body mass index								
Global	4.99 (2.15–8.04)	4.90 (2.15 –7.70)	−0.25 (−0.35– −0.16)	< .001	125.74 (54.27–202.56)	125.23 (54.82–197.46)	−0.20 (−0.28– −0.11)	< .001
High SDI	9.09 (3.90–14.72)	7.07 (3.09–11.11)	−0.99 (−1.06– −0.92)	< .001	230.99 (99.37–374.02)	184.01 (80.31–290.15)	−0.90 (−0.97– −0.83)	< .001
High-middle SDI	6.86 (2.95–11.06)	7.38 (3.28–11.69)	−0.06 (−0.21–0.09)	.442	171.96 (73.81–277.51)	188.37 (83.52–297.24)	0.01 (−0.13–0.16)	.844
Middle SDI	1.80 (0.68–2.92)	3.54 (1.52–5.65)	2.22 (2.19–2.25)	< .001	44.74 (16.84–72.75)	89.65 (38.28 –142.06)	2.28 (2.25–2.32)	< .001
Low-middle SDI	0.94 (0.36–1.51)	2.07 (0.86–3.29)	2.76 (2.69–2.83)	< .001	23.32 (9.05–37.54)	51.61 (21.52–81.84)	2.78 (2.71–2.85)	< .001
Low SDI	0.97 (0.36–1.64)	1.44 (0.55–2.32)	1.18 (1.09–1.27)	< .001	23.95 (8.81–40.59)	35.60 (13.57–57.32)	1.19 (1.09–1.28)	< .001
High fasting plasma glucose								
Global	3.79 (1.88–5.67)	4.04 (2.05–6.11)	0.18 (0.10–0.25)	< .001	95.24 (47.45–142.48)	103.26 (52.12–156.44)	0.23 (0.17–0.30)	< .001
High SDI	5.86 (2.87–8.55)	5.41 (2.66–8.07)	−0.45 (−0.58– −0.31)	< .001	149.07 (72.80–219.18)	140.89 (70.32–209.79)	−0.36 (−0.50– −0.22)	< .001
High-middle SDI	4.44 (2.22–6.77)	4.98 (2.55–7.51)	0.43 (0.28–0.58)	< .001	111.24 (55.64–169.43)	127.24 (65.10–192.53)	0.51 (0.37–0.65)	< .001
Middle SDI	2.64 (1.32–4.06)	3.47 (1.76–5.31)	1.00 (0.93–1.07)	< .001	65.60 (32.96–100.96)	87.67 (44.19–134.49)	1.06 (0.99–1.13)	< .001
Low-middle SDI	1.48 (0.72–2.26)	2.63 (1.28–4.09)	1.87 (1.80–1.94)	< .001	36.75 (17.87–56.02)	65.33 (31.94–101.87)	1.88 (1.81–1.96)	< .001
Low SDI	1.61 (0.80–2.56)	1.96 (0.92–2.98)	0.51 (0.41–0.61)	< .001	39.85 (19.80–63.42)	48.60 (22.87–73.90)	0.52 (0.41–0.62)	< .001
Diet high in red meat								
Global	9.31 (0.00–18.50)	7.09 (0.00–14.34)	−1.03 (−1.10– −0.96)	< .001	234.25 (0.09–468.29)	181.38(0.08–366.06)	−0.97 (−1.03– −0.90)	< .001
High SDI	14.47 (0.01–28.51)	9.04 (0.00–17.99)	−1.68 (−1.77– −1.60)	< .001	368.04 (0.19–726.94)	235.68 (0.12–464.46)	−1.59 (−1.67– −1.52)	< .001
High-middle SDI	12.03 (0.00–24.17)	9.98 (0.01–20.26)	−0.80 (−0.91– −0.69)	< .001	301.19 (0.10–604.07)	255.37 (0.13–518.06)	−0.72 (−0.81– −0.62)	< .001
Middle SDI	6.08 (0.00–12.50)	6.30 (0.00–12.77)	0.13 (0.07–0.18)	< .001	151.01 (0.02–310.13)	159.41 (0.06–324.62)	0.20 (0.14–0.26)	< .001
Low-middle SDI	2.45 (0.00–5.16)	3.04 (0.00–6.24)	0.75 (0.72–0.79)	< .001	60.70 (0.01–127.85)	75.75 (0.01–155.51)	0.77 (0.73–0.81)	< .001
Low SDI	3.71 (0.00–7.93)	3.07 (0.00–6.26)	−0.69 (−0.78– −0.61)	< .001	91.55 (0.02–196.07)	76.03 (0.01–154.98)	−0.69 (−0.77– −0.60)	< .001
Diet high in processed meat								
Global	4.18 (1.03–8.39)	2.59 (0.62–5.27)	−1.66 (−1.76– −1.57)	< .001	105.85 (26.19–213.19)	66.71 (16.00–136.59)	−1.60 (−1.69– −1.50)	< .001
High SDI	8.74 (2.17–17.66)	5.47 (1.37–10.90)	−1.57 (−1.67– −1.46)	< .001	222.54 (55.42–450.21)	142.80 (35.80–285.53)	−1.48 (−1.59– −1.37)	< .001
High-middle SDI	5.42 (1.37–10.92)	3.70 (0.88–7.64)	−1.63 (−1.86– −1.4)	< .001	136.20 (34.46–274.97)	94.52 (22.47–195.17)	−1.56 (−1.78– −1.34)	< .001
Middle SDI	0.72 (0.17–1.50)	1.03 (0.21–2.20)	1.45 (1.33–1.58)	< .001	17.86 (4.13–37.07)	26.07 (5.34–55.17)	1.52 (1.39–1.65)	< .001
Low-middle SDI	0.57 (0.14–1.21)	0.71 (0.16–1.50)	0.82 (0.76–0.88)	< .001	14.00 (3.58–30.06)	17.63 (3.90–37.21)	0.84 (0.78–0.90)	< .001
Low SDI	1.00 (0.24–2.23)	0.89 (0.21–1.92)	−0.46 (−0.54– −0.38)	< .001	24.67 (5.87–55.10)	22.16 (5.24–47.46)	−0.45 (−0.53– −0.37)	< .001

Diet low in fiber								
Global	0.87 (0.40–1.40)	0.50 (0.22–0.80)	2.00 (−2.1– −1.9)	< .001	21.70 (10.11–35.04)	12.58 (5.54–20.31)	−1.94 (−2.04– −1.85)	< .001
High SDI	1.10 (0.49–1.69)	0.54 (0.24–0.85)	−2.28 (−2.33– −2.23)	< .001	28.03 (12.54–43.24)	14.12 (6.24–22.24)	−2.18 (−2.22– −2.13)	< .001
High-middle SDI	0.69 (0.30–1.18)	0.41 (0.18–0.74)	−1.97 (−2.23– −1.7)	< .001	17.20 (7.59–29.70)	10.51 (4.54–18.87)	−1.88 (−2.13– −1.63)	< .001
Middle SDI	0.96 (0.41–1.57)	0.56 (0.25–0.90)	−2.01 (−2.11– −1.9)	< .001	23.87 (10.22–38.89)	14.00 (6.30–22.61)	−1.96 (−2.06– −1.86)	< .001
Low-middle SDI	0.77 (0.34–1.22)	0.52(0.22–0.83)	−1.69 (−1.87– −1.5)	< .001	19.04 (8.44–30.18)	12.92(5.48–20.56)	−1.68 (−1.86– −1.49)	< .001
Low SDI	0.29 (0.13–0.46)	0.24 (0.10–0.39)	−0.97 (−1.31– −0.63)	< .001	7.26 (3.21–11.45)	5.95 (2.56–9.65)	−0.97 (−1.3– −0.63)	< .001
Diet low in calcium								
Global	5.93 (4.44–7.45)	4.00 (2.92–5.02)	−1.49 (−1.58– −1.39)	< .001	147.90 (110.58–184.93)	101.09 (73.86–126.60)	−1.44 (−1.53– −1.35)	< .001
High SDI	3.45 (2.41–4.58)	1.98 (1.38–2.65)	−1.84 (−1.90– −1.78)	< .001	87.67 (61.73–116.67)	51.67 (36.15–69.21)	−1.75 (−1.8– −1.69)	< .001
High-middle SDI	5.72 (4.05–7.38)	3.36 (2.36–4.44)	−1.99 (−2.12– −1.86)	< .001	142.69 (101.22–184.48)	85.76 (60.54–113.72)	−1.90 (−2.02– −1.77)	< .001
Middle SDI	8.31 (6.13–10.28)	5.17 (3.75–6.53)	−1.70 (−1.77– −1.62)	< .001	206.11 (152.11–255.75)	130.14 (94.26–164.57)	−1.65 (−1.72– −1.57)	< .001
Low-middle SDI	5.66 (4.23–7.31)	4.62 (3.35–5.68)	−0.84 (−0.95– −0.73)	< .001	140.14 (104.58–181.01)	114.78 (83.13–141.46)	−0.83 (−0.94– −0.72)	< .001
Low SDI	9.23 (6.67–11.83)	6.25 (4.63–7.80)	−1.57 (−1.68– −1.46)	< .001	227.97 (164.54–292.41)	154.76 (115.01–193.74)	−1.57 (−1.68– −1.46)	< .001
Diet low in milk								
Global	8.68 (2.41–14.28)	7.21 (1.99–11.95)	−0.71 (−0.80– −0.62)	< .001	217.58 (60.51–356.83)	183.55 (50.37–304.58)	−0.65 (−0.74– −0.56)	< .001
High SDI	10.56 (2.86–17.69)	6.45 (1.71–10.64)	−1.82 (−1.93– −1.7)	< .001	268.40 (72.52–447.20)	167.96 (44.06–277.06)	−1.73 (−1.84– −1.62)	< .001
High-middle SDI	9.67 (2.66–16.19)	8.74 (2.40–14.82)	−0.29 (−0.39– −0.19)	< .001	241.74 (66.41–405.68)	223.83 (61.33–378.57)	−0.20 (−0.30– −0.09)	.001
Middle SDI	8.25 (2.19–13.45)	7.78 (2.11–12.89)	−0.25 (−0.31– −0.19)	< .001	204.80 (54.35–333.79)	196.72 (53.15–323.87)	−0.18 (−0.24– −0.12)	< .001
Low-middle SDI	4.92 (1.41–8.13)	5.42 (1.50–8.87)	0.26 (0.19–0.34)	< .001	121.83 (34.83–201.33)	134.57 (37.31–219.84)	0.28 (0.20–0.35)	< .001
Low SDI	6.49 (1.73–10.82)	5.41 (1.49–8.76)	−0.75 (−0.83– −0.66)	< .001	160.37 (42.84–267.17)	133.96 (36.97–217.19)	−0.74 (−0.83– −0.65)	< .001
Low physical activity								
Global	3.42 (2.09–4.76)	2.62 (1.57–3.71)	−0.99 (−1.08– −0.91)	< .001	86.06 (52.64–119.98)	66.96 (40.47–94.14)	−0.93 (−1.02– −0.85)	< .001
High SDI	5.35 (3.05–7.79)	3.20 (1.84–4.57)	−1.78 (−1.88– −1.67)	< .001	136.01 (77.51–198.26)	83.51 (48.15–118.95)	−1.68 (−1.78– −1.58)	< .001
High-middle SDI	3.76 (2.32–5.38)	3.25 (1.91–4.88)	−0.60 (−0.65– −0.54)	< .001	94.07 (58.18–134.99)	83.23 (49.07–124.68)	−0.51 (−0.56– −0.46)	< .001
Middle SDI	2.56 (1.51–3.85)	2.53(1.48–3.76)	−0.17 (−0.24– −0.10)	< .001	63.54 (37.68–95.42)	63.94 (37.22–95.14)	−0.11 (−0.18– −0.03)	.006
Low-middle SDI	1.48 (0.93–2.18)	1.61 (0.95–2.32)	0.12 (0.01–0.23)	.033	36.61 (22.95–53.91)	40.04 (23.68–57.78)	0.14 (0.03–0.25)	.017
Low SDI	1.24 (0.71–1.87)	1.02 (0.60–1.49)	−0.82 (−0.90– −0.74)	< .001	30.59 (17.68–46.30)	25.17 (14.77–37.08)	−0.82 (−0.89– −0.74)	< .001
Smoking								
Global	4.03 (2.55–5.42)	2.74 (1.72–3.79)	−1.38 (−1.45– −1.30)	< .001	101.31 (64.19–136.68)	69.92 (43.80–96.99)	−1.32 (−1.40– −1.24)	< .001
High SDI	6.21 (3.96–8.47)	3.10 (1.91–4.30)	−2.44 (−2.53– −2.34)	< .001	157.90 (101.00–216.09)	80.69 (49.69–112.13)	−2.35 (−2.44– −2.27)	< .001
High-middle SDI	4.76 (3.06–6.50)	3.92 (2.44–5.53)	−0.75 (−0.85– −0.66)	< .001	119.27 (76.80–162.77)	100.27 (62.47–141.17)	−0.67 (−0.76– −0.58)	< .001

Middle SDI	2.99 (1.90–4.10)	2.63 (1.63–3.71)	−0.37 (−0.48– −0.26)	< .001	74.29 (47.30–101.89)	66.49 (41.39–94.18)	−0.30 (−0.41– −0.19)	< .001
Low-middle SDI	1.50 (0.92–2.13)	1.36 (0.85–1.94)	−0.39 (−0.46– −0.31)	< .001	37.18 (22.81–52.61)	33.81 (21.14–48.10)	−0.37 (−0.45– −0.30)	< .001
Low SDI	1.06 (0.64–1.56)	0.76 (0.45–1.08)	−1.22 (−1.32– −1.12)	< .001	26.18 (15.75–38.43)	18.89 (11.28–26.85)	−1.22 (−1.32– −1.12)	< .001
High alcohol use								
Global	3.89 (2.97–4.80)	2.81 (2.24–3.50)	−1.17 (−1.23– −1.1)	< .001	98.15 (74.92–121.42)	72.17 (57.32–89.70)	−1.10 (−1.16– −1.04)	< .001
High SDI	7.28 (5.44–9.17)	4.64 (3.70–5.59)	−1.63 (−1.74– −1.53)	< .001	185.12 (138.23–233.34)	120.83 (96.35–145.59)	−1.54 (−1.64– −1.45)	< .001
High-middle SDI	5.21 (3.91–6.49)	4.12 (3.16–5.19)	−0.93 (−1.08– −0.78)	< .001	130.72 (97.87–162.71)	105.37 (80.33–133.25)	−0.85 (−0.99– −0.71)	< .001
Middle SDI	1.53 (1.11–2.01)	2.00 (1.46–2.66)	1.08 (0.87–1.30)	< .001	38.07 (27.61–49.90)	50.57 (37.07–66.83)	1.16 (0.93–1.38)	< .001
Low-middle SDI	0.41 (0.28–0.55)	0.67 (0.49–0.88)	1.89 (1.72–2.06)	< .001	10.12 (7.05–13.56)	16.58 (12.17–21.80)	1.90 (1.73–2.07)	< .001
Low SDI	0.71 (0.44–0.96)	0.82 (0.58–1.11)	0.33 (0.01–0.65)	.043	17.59 (10.96–23.86)	20.36 (14.39–27.40)	0.34 (0.01–0.66)	.041

CI = confidence interval, DALYs = disability adjusted life years, EAPC = estimated annual percentage change, SDI = socio-demographic index, UI = uncertainty interval.

Table S2

Predicted age-standardized incidence, prevalence, mortality, and DALYs rates of colorectal cancer among individuals aged 65–74 years from 2022 to 2050, based on results from the autoregressive integrated moving average model.

Characteristics	Age-standardized incidence rate				Age-standardized prevalence rate				Age-standardized mortality rate				Age-standardized DALYs rate			
	2022 (95% UI)	2050 (95% UI)	2022–2050 EAPC (95% CI)	*P* value	2022 (95% UI)	2050 (95% UI)	2022–2050 EAPC (95% CI)	*P* value	2022 (95% UI)	2050 (95% UI)	2022–2050 EAPC (95% CI)	*P* value	2022 (95% UI)	2050 (95% UI)	2022–2050 EAPC (95% CI)	*P* value
Global	137.11 (135.52–138.70)	133.24 (126.26–140.23)	−0.07 (−0.12– −0.03)	.001	763.51 (756.5–770.52)	894.15 (659.34–1128.97)	0.57 (0.56–0.57)	< .001	58.43 (57.56–59.30)	46.92 (35.00–58.84)	−0.80 (−0.82– −0.78)	< .001	1353.26 (1334.03–1372.50)	1103.45 (844.06–1362.85)	−0.74 (−0.76– −0.73)	< .001
Sex																
Female	103.83 (102.38–105.29)	105.57 (86.60–124.53)	0.03 (0.01–0.04)	.001	589.97 (583.38–596.56)	652.50 (592.86 –712.14)	0.35 (0.35–0.35)	< .001	44.53 (43.75 –45.30)	31.02 (20.01 –42.03)	−1.32 (−1.36– −1.28)	< .001	1029.04 (1011.85 –1046.24)	730.83 (488.91 –972.76)	−1.25 (−1.29– −1.21)	< .001
Male	174.49 (172.43–176.56)	202.62 (86.78–318.46)	0.53 (0.53–0.54)	< .001	958.71 (950.44–966.99)	1120.33 (866.39–1374.26)	0.56 (0.55–0.56)	< .001	74.11 (72.97–75.24)	74.06 (55.91–92.21)	0.00 (0.00–0.00)	< .001	1717.84 (1692.74 –1742.93)	1714.72 (1325.46–2103.97)	0.00 (0.00–0.00)	< .001
GBD regions																
Andean Latin America	80.05 (77.31–82.78)	104.29 (85.00–123.57)	0.95 (0.93–0.96)	< .001	432.56 (418.24–446.88)	621.75 (484.54–758.96)	1.32 (1.30–1.34)	< .001	49.67 (47.77–51.58)	49.67 (39.40–59.94)	0.00 (0.00–0.00)	.095	1128.69 (1087.08–1170.31)	1128.69 (838.37–1419.02)	0.00 (−0.00–0.00)	.095
Australasia	213.4 (202.49–224.31)	190.96 (0.00–633.23)	−0.40 (−0.40– −0.39)	< .001	1415.95 (1350.08–1481.82)	1252.53 (0.00–3895.37)	−0.44 (−0.44– −0.43)	< .001	59.90 (56.53–63.26)	1.13 (0.00–19.24)	−9.52 (−11.18– −7.84)	< .001	1432.46 (1353.96–1510.97)	113.28 (0.00–536.06)	−7.37 (−8.23– −6.50)	< .001
Caribbean	198.69 (194.7–202.67)	256.72 (235.28–278.16)	0.92 (0.91–0.93)	< .001	1036.58 (1016.11–1057.06)	1448.77 (1338.5–1559.05)	1.20 (1.18–1.22)	< .001	71.21 (69.79–72.64)	71.21 (63.54–78.88)	0.00 (0.00–0.00)	.095	1661.61 (1627.66–1695.56)	1661.61 (1478.78–1844.43)	0.00 (0.00–0.00)	.095

Central Asia	65.03 (62.42–67.64)	65.74 (59.32–72.17)	0.03 (0.02–0.03)	< .001	255.67 (246.28–265.06)	285.2 (8.12–562.27)	0.33 (0.31–0.35)	< .001	45.88 (44.10–47.65)	35.91 (28.29–43.53)	−0.90 (−0.92– −0.88)	< .001	1041.68 1001.55–1081.81)	807.07 (625.87–988.27)	−0.93 (−0.95– −0.92)	< .001
Central Europe	233.57 (229.03–238.1)	261.21 (124.98–397.45)	0.40 (0.40–0.40)	< .001	1136.37 (1118.68–1154.07)	1347.76 (850.82–1844.7)	0.61 (0.61–0.62)	< .001	124.63 (121.84 –127.42)	125.14 (114.44–135.83)	0.01 (0.01–0.01)	< .001	2852.52 2793.38–2911.67)	2867.17 (2648.45–3085.89)	0.01 (−0.01–0.04)	.320
Central Latin America	101.57 (99.29–103.86)	147.12 (134.8–159.44)	1.33 (1.30–1.35)	< .001	475.11 (465.00–485.23)	835.26 (623.53–1046.99)	2.02 (1.96–2.07)	< .001	46.09 (45.02–47.17)	56.68 (50.91–62.45)	0.74 (0.73–0.75)	< .001	1062.70 (1038.13–1087.27)	1321.12 (1188.81–1453.43)	0.78 (0.77–0.79)	< .001
Central Sub−Saharan Africa	42.99 (42.38–43.59)	50.15 (23.46–76.85)	0.55 (0.55–0.56)	< .001	124.74 (123.18–126.29)	165.17 (86.12–244.22)	1.01 (0.99–1.02)	< .001	39.37 (38.83–39.92)	38.51 (35.71–41.31)	−0.07 (−0.09– −0.05)	< .001	890.39 (878.35–902.42)	868.73 (804.21–933.25)	−0.09 (−0.12– −0.07)	< .001
East Asia	163.76 (161.49–166.04)	228.10 (206.73–249.48)	1.19 (1.17–1.21)	< .001	923.75 (910.93–936.58)	1574.58 (1024.51–2124.65)	1.91 (1.86–1.96)	< .001	63.65 (62.35–64.95)	63.65 (51.00–76.30)	−0.00 (−0.00–0.00)	.095	1479.20 1450.01–1508.39)	1479.20 (1196.42–1761.98)	−0.00 (−0.00–0.00)	.095
Eastern Europe	194.82 (187.26–202.38)	233.40 (192.70–274.10)	0.65 (0.64–0.65)	< .001	930.22 (901.65–958.79)	1218.15 (1064.28–1372.01)	0.97 (0.95–0.98)	< .001	102.77 (98.00–107.54)	102.77 (77.07–128.47)	0.00 (0.00–0.00)	.095	2362.81 (2255.87–2469.74)	2362.81 (1786.95–2938.67)	−0.00 (−0.00–0.00)	.095
Eastern Sub−Saharan Africa	57.6 (57.01–58.19)	68.31 (34.06–102.57)	0.61 (0.61–0.62)	< .001	169.14 (167.03–171.26)	224.06 (154.81–293.32)	1.01 (0.99–1.02)	< .001	52.05 (51.50–52.61)	53.96 (21.80–86.12)	0.13 (0.13–0.13)	< .001	1172.70 (1160.91 – 1184.49)	1258.07 (569.14 – 1946.99)	0.25 (0.25–0.25)	< .001
High−income Asia Pacific	256.64 (248.8–264.48)	283.36 (103.48–463.23)	0.35 (0.35–0.36)	< .001	1710.98 (1664.11–1757.84)	1997.26 (926.96–3067.56)	0.55 (0.55–0.56)	< .001	71.49 (69.37–73.62)	71.49 (50.65–92.33)	0.00 (0.00–0.00)	.095	1702.76 (1653.80 – 1751.72)	1702.76 (1235.68 – 2169.84)	−0.00 (−0.00–0.00)	.095
High−income North America	186.11	294.45	1.64	< .001	1207.82	1897.15	1.62 (1.58–1.65)	< .001	54.83	11.23	−5.14	< .001	1308.46	314.54	−4.69	< .001

	(179.43–192.79)	(0.00–912.63)	(1.60–1.68)		(1169.05–1246.58)	(0.00–5482.87)			(52.92–56.75)	(0.00–26.75)	(−5.53– −4.74)		(1264.62–1352.30)	(0.00–668.72)	(−5.01– −4.37)	
North Africa and Middle East	74.39 (73.58–75.20)	96.25 (82.14–110.37)	0.94 (0.93–0.94)	< .001	401.47 (398.13–404.81)	568.8 (486.99–650.60)	1.28 (1.27–1.29)	< .001	43.60 (43.03–44.17)	43.18 (33.20–53.16)	−0.02 (−0.03– −0.01)	< .001	998.55 (985.42–1011.67)	989.03 (756.66–1221.39)	−0.02 (−0.03– −0.01)	< .001
Oceania	31.65 (31.17–32.13)	31.65 (28.00–35.30)	0.00 (0.00–0.00)	.095	104.98 (103.20–106.76)	104.85 (88.02–121.67)	−0.00 (−0.00– −0.00)	.010	26.27 (25.88–26.66)	23.97 (20.97–26.97)	−0.33 (−0.33– −0.33)	< .001	596.26 (587.65–604.88)	545.02 (478.12–611.91)	−0.32 (−0.32– −0.32)	< .001
South Asia	30.33 (29.38–31.28)	30.37 (20.82–39.93)	0.00 (0.00–0.00)	.009	101.13 (98.6–103.67)	129.27 (100.79–157.75)	0.89 (0.88–0.90)	< .001	24.40 (23.63–25.17)	23.82 (22.19–25.45)	−0.02 (−0.04– −0.00)	.054	553.39 (536.14–570.64)	539.66 (502.23–577.09)	−0.02 (−0.05–0.00)	.062
Southeast Asia	95.18 (94.23–96.12)	127.41 (122.32–132.50)	1.04 (1.03–1.06)	< .001	383.13 (379.75–386.5)	559.51 (532.48–586.55)	1.36 (1.33–1.38)	< .001	65.80 (65.12–66.49)	73.62 (51.12–96.13)	0.40 (0.40–0.40)	< .001	1498.19 (1482.55 – 1513.84)	1701.29 (1234.27 – 2168.30)	0.45 (0.45–0.46)	< .001
Southern Sub−Saharan Africa	66.63 (64.09–69.17)	63.17 (2.56–123.78)	−0.19 (−0.19– −0.19)	< .001	219.94 (213.53–226.35)	296.12 (261.6–330.63)	1.06 (1.05–1.08)	< .001	53.81 (51.51–56.10)	40.42 (0.00–109.1)	−1.01 (−1.03– −1.00)	< .001	1222.22 (1170.02–1274.41)	936.70 (0.00–2502.71)	−0.94 (−0.96– −0.93)	< .001
Tropical Latin America	92.07 (89.91–94.22)	122.37 (110.77–133.96)	1.02 (1.00–1.03)	< .001	403.47 (395.08–411.87)	585.46 (540.25–630.67)	1.33 (1.31–1.36)	< .001	57.34 (55.90–58.78)	58.90 (32.80–85.00)	0.10 (0.10–0.10)	< .001	1308.45 (1275.85–1341.05)	1373.71 (804.33–1943.1)	0.17 (0.17–0.17)	< .001
Western Europe	218.62 (212.54–224.70)	141.73 (0.00–460.82)	−1.53 (−1.56– −1.49)	< .001	1406.45 (1373.50–1439.4)	934.64 (0.00–2151.76)	−1.44 (−1.47– −1.41)	< .001	68.94 (67.11–70.76)	33.39 (17.19–49.60)	−2.53 (−2.62– −2.43)	< .001	1634.31 (1593.71–1674.92)	852.41 (506.20–1198.61)	−2.28 (−2.35– −2.20)	< .001
Western Sub−Saharan Africa	32.51 (32.12–	32.41 (25.40–	−0.01 (−0.01– −0.01)	< .001	94.46 (93.38–	105.08 (48.41–	0.38 (0.38–0.38)	< .001	29.10 (28.74–	28.68 (23.37–	−0.03 (−0.04– −0.02)	< .001	654.02 (646.27–	646.17 (530.84–	−0.03 (−0.04– −0.02)	< .001

32.89)	39.43)		95.55)	161.75)	29.46)	34.00)	661.78)	761.50)

CI = confidence interval, DALYs = disability adjusted life years, EAPC = estimated annual percentage change, GBD = Global Burden of Disease, UI = uncertainty interval.
